# NorthEuraLex: a wide-coverage lexical database of Northern Eurasia

**DOI:** 10.1007/s10579-019-09480-6

**Published:** 2019-11-30

**Authors:** Johannes Dellert, Thora Daneyko, Alla Münch, Alina Ladygina, Armin Buch, Natalie Clarius, Ilja Grigorjew, Mohamed Balabel, Hizniye Isabella Boga, Zalina Baysarova, Roland Mühlenbernd, Johannes Wahle, Gerhard Jäger

**Affiliations:** Seminar für Sprachwissenschaft, Universität Tübingen, Wilhelmstraße 19, 72074 Tübingen, Germany

**Keywords:** Lexical database, Northern Eurasia, Indo-European languages, Uralic languages, Turkic languages, Siberian languages, Caucasian languages

## Abstract

This article describes the first release version of a new lexicostatistical database of Northern Eurasia, which includes Europe as the most well-researched linguistic area. Unlike in other areas of the world, where databases are restricted to covering a small number of concepts as far as possible based on often sparse documentation, good lexical resources providing wide coverage of the lexicon are available even for many smaller languages in our target area. This makes it possible to attain near-completeness for a substantial number of concepts. The resulting database provides a basis for rich benchmarks that can be used to test automated methods which aim to derive new knowledge about language history in underresearched areas.

## Introduction

The most basic prerequisite for any computational study in historical linguistics is an electronic database which contains the information a linguist would look up in dictionaries or other sources in a machine-readable format. The absence of such databases has been one of the limiting factors to the development of the field, but some very useful resources have become available during the past decade (Dunn 2015; Greenhill et al. 2008; Wichmann et al. 2016), and the pace at which new resources appear is accelerating (Greenhill 2015; Bowern 2016; Kaiping and Klamer 2018).

While the number of large-scale lexical databases is increasing, most of them systematically cover only about 100 or 200 very basic concepts for each language. An extreme case among these narrow-coverage databases is the database produced by the Automated Similarity Judgment Program (ASJP) (Wichmann et al. 2016), which due to its global coverage of more than 5000 languages is by far the largest in terms of the number of languages covered, but only includes data for a mere 40 concepts per language. The rationale for this reductionist approach is presented in Holman et al. (2008), arguing that longer lists do not improve performance in simple language classification and phylogenetic inference. Beyond these tasks, the ASJP database has been used to investigate many issues of general interest to historical linguists, like the stability of concepts against borrowing and semantic change, the question whether sound symbolism creates problematic amounts of lexical similarity between unrelated languages, and whether there are correlations between phoneme inventory sizes and extralinguistic factors such as population size or geographic isolation.

While it is of course much more feasible to achieve a good global coverage with such short lists, more complex tasks in tools for computational historical linguistics tend to require more data. Such tasks include the extraction of regular sound correspondences as are necessary to actually prove language relationships, the automated detection of loanwords or cognates which have undergone semantic shifts, and models for detecting contact events between languages.

Some interesting datasets have arisen from studies which investigate automated approaches to such tasks. For instance, the Austronesian Basic Vocabulary Database (ABVD) by Greenhill et al. (2008) was used by Bouchard-Côté et al. (2013) to evaluate their system for Bayesian reconstruction of proto-forms, and the IELex database of Indo-European basic vocabulary (Dunn 2015) grew out of an early lexicostatistical study by Dyen et al. (1992). Both databases primarily provide cognacy annotations which have been used for phylogenetic inference, but use either the official orthographies or often idiosyncratic transliterations instead of providing a phonetic transcription for those languages which have a standardized written form. At 1400 languages, the ABVD provides virtually complete coverage of the world’s largest language family, and IELex is popular due to the central role of Indo-European in historical linguistics. Both ABVD and IELex cover lists of about 200 concepts, which is substantially more than the ASJP database, but far from enough for work on regular sound correspondences. Moreover, since each of these databases only covers a single language family, they are not adequate for the study of cross-family patterns.

More comprehensive lexical resources which aim to cover a substantial part of the basic vocabulary across more than one language family, have recently started to appear for some linguistic areas in the world. For instance, TransNewGuinea.org (Greenhill 2015) includes data about more than 800 languages and dialects of New Guinea, the least well-studied linguistic region of the world, and aims to provide a unified phonetic format that can be processed by computational tools. Due to the very sparse documentation of many languages, the total size of this database will not be able expand far beyond its current 125,000 entries, or an average of just over 150 words per language. The Chirila database of Australian languages by Bowern (2016) is similar in scope and structure, with the goal of eventually making all known lexical data available. Due to the complicated legal situation when publishing full resources, and history-induced hesitancy of many linguistic groups when it comes to giving outsiders access to their languages, only 150,000 of 780,000 database entries are freely available at the moment. The LexiRumah database by Kaiping and Klamer (2018) summarizes the result of extensive fieldwork on the languages of the Indonesian islands of Alor and Pantar, many of which are Papuan (i.e. non-Austronesian) languages, with an average of 337 concepts across 101 language varieties.

Currently, no comparable cross-family database exists for any of the more well-researched linguistic areas in the world. The only wide-coverage lexical database which covers a multitude of language families from different regions is the Intercontinental Dictionary Series (IDS) edited by Key and Comrie (2015). This collection of dictionaries has the advantage of consisting of expert contributions, but has not been extended for years, and remains at just over 329 different languages from all over the world, with a focus on some families like Nakh-Daghestanian, Tai-Kadai, and Austroasiatic. The disadvantages of this database are that it does not cover any larger geographical area which could be used for cross-family contact models, and that there are large gaps in lexical coverage even for languages where more complete resources would be available.

NorthEuraLex, the preliminary release version of which we present in this article, currently spans a large list of 1016 concepts across 107 languages predominantly from Northern Eurasia. Across the total of 121,614 dictionary forms contained in the database, a uniform phonetic transcription in IPA is available. The vast majority of these transcriptions has been generated automatically based on phonological descriptions for the languages, except for about a dozen languages whose complex historically grown orthographies did not allow for that (e.g. English, French, Danish, and Irish), or whose writing systems do not completely represent the phonology (e.g. Chinese, Japanese, and Persian). For some of these languages, like Arabic and Hebrew, we are able to make use of extended dictionary orthographies which provide full information about pronunciation. Because a consistent IPA transcription is used for all of our word lists, they can be converted automatically into a range of other less fine-grained transcription formats, such as ASJP encoding, in order to combine them with other resources.

NorthEuraLex occupies a unique position in a fast-growing landscape of basic vocabulary databases which by now cover many linguistic areas. Due to the scarcity of documentation and unfeasibility of fieldwork covering hundreds of languages for a single institution that could ensure a uniform treatment, most of the other databases will always remain gappy. Northern Eurasia is very different in this respect, because the vast majority of its languages is well-documented, making it possible to reach near-complete coverage across an entire linguistic area. The studies building on the ASJP database exemplify the type of research that will be facilitated with the availability of our wide-coverage cross-family database in a uniform transcription format.

The remainder of this paper is divided intro three main sections. The purpose of Sect. 2 is to describe and motivate our choices for the languages we included, and the procedure by which we arrived at our concept list. Our technical design decisions as well as the data collection procedure are detailed in Sect. 3, which also includes a general description of our approach to generating IPA transcriptions. Section 4 describes our initial evaluation of data quality for a sample of six languages with the help of native speakers, discussing and providing figures on the frequency of different types of errors. The paper concludes with an overview of our ongoing expansion efforts as well as plans for future development.

## Scope

### Language sample

The focus of NorthEuraLex lies on Northern Eurasia, more precisely on the Uralic family and surrounding languages. A prime reason for this choice is that these languages have already been extensively documented and analyzed, so there are enough sources available to find equivalents of the concepts in our long list largely without the help of experts or native speakers. The comparatively small size of Uralic as opposed to e.g. Indo-European, as well as the limited number of language families which have historically been in contact with Uralic, makes it feasible to achieve complete coverage for Uralic and all relevant neighboring families. Having a more or less complete sample of a family and its contact languages provides an ideal data set for computational models of lexical influence, which has been the primary use case of the database within our project.

**Fig. 1:**
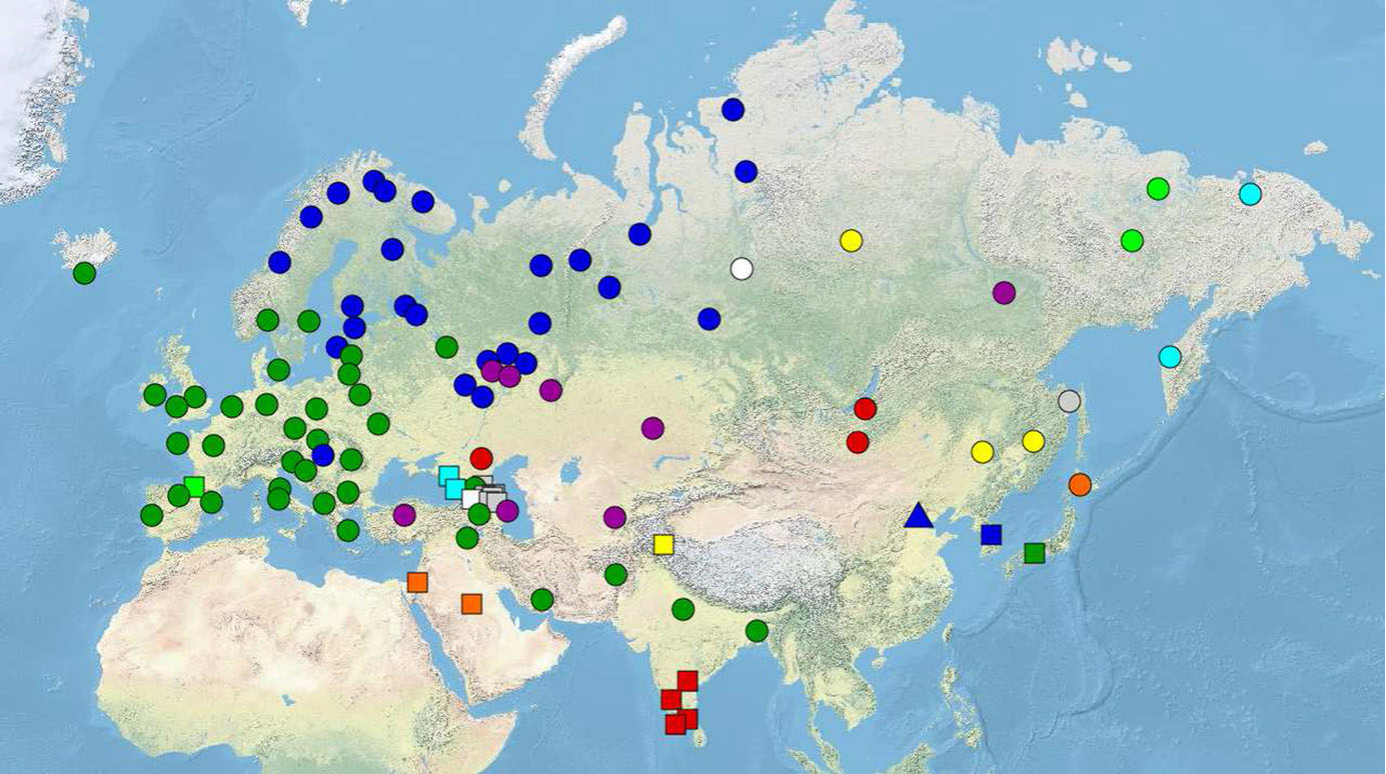
Map of languages in NorthEuraLex 0.9 (except Eskimo-Aleut). Each language family is encoded by a different combination of color and shape. (Color figure online)

In addition to all the 26 Uralic languages for which sufficient lexical resources were initially available to us, we have collected data for all language families of the area, which includes Indo-European, Turkic, Mongolic, Tungusic, Korean, Japanese as well as all the five Paleo-Siberian and the three Caucasian families, plus prominent isolates like Basque or Burushaski. In the course of the data collection process, we also started to include samples from some adjacent families outside Northern Eurasia, such as Dravidian, Afro-Asiatic, and Eskimo–Aleut. Overall, our first release version contains data for 107 languages from 21 families, which we are in the process of expanding substantially over the coming years. Table 2 in Appendix A lists all the languages included in NorthEuraLex 0.9, and our coverage of the Eurasian continent is visualized (with one symbol per language family) in Fig. 1. This initial choice of languages represents a pragmatic sample based on giving preference to large languages for which dictionaries are readily available, as well as to particularly interesting languages like language isolates or members of very small families. We estimate that a total of 350 languages and language varieties could (and are intended to) eventually be included in NorthEuraLex.

### Concept selection

To decide for which concepts to collect data, we could have adopted one of two extant long lists of basic concepts, i.e. the list used by the IDS dictionaries, or the Loanword Typology meaning list of the World Loanword Database (Haspelmath and Tadmor 2009), which is designed to find patterns of borrowability in a range of different semantic domains. The problem with both lists is that they include many concepts which are not current to indigenous cultures of our area, or at least difficult to find in the available lexical resources. This would have compromised our goal of a gapless database too much. Moreover, the two lists are only very distantly and informally grounded in data. Tracing back their history, one finds that the WOLD list is an extended version of the IDS list, which in turn is a revision of a list developed by Buck (1949) for the purpose of tracing the diachrony of Indo-European synonyms. The two existing long concept lists are therefore not only not independent, but they are very likely to share a bias towards including concepts which are of specific relevance to Indo-European languages only.

For these reasons, our database is based on a new concept list which has an empirical basis in a subset of the relevant languages. In the initial stage of the data collection project, we used a pre-existing private database of about 20,000 entries each from 12 major languages of Eurasia (with German as the common gloss language) which were collected by the first author for the purposes of language learning, along with data from a sample of five languages for which only dictionaries from a series of Soviet-era school dictionaries are available. These dictionaries, such as Menovšc̆ikov (1988) for Siberian Yupik and Volodin and Halojmova (1989) for Itelmen, are sometimes the only published lexical resource for the languages in question. Based on these data, our task was to choose a list of 1000 concepts that are both old enough to be of value for historical linguistics and relevant for the geographical area, while still being realistically extractable from available dictionaries.

On this initial dataset, we computed an early version of the basicness score later presented in Dellert and Buch (2018), which combines a cross-linguistically applicable measure of form simplicity with a form-distance based measure of stability. While the form simplicity measure could be applied to any orthography which encodes pronunciation, our form distance measure presupposes uniform encoding across languages, and we derive it from automatically derived phonemic IPA transcriptions. The simplicity measure computes the information content of each dictionary form in a language-specific way, generalizing word length by correcting for differences in sound systems, and morphological material in the dictionary forms. The stability measure builds on a definition of pairwise word form distances that takes some types of sound correspondences into account, and is an estimate of the correlation between the pairwise distances between the realizations of the concept in question and the aggregate language distances across all concepts.

From the top-1000 list in the resulting rough ranking of about 6000 concepts described by sets of German lemmas, we manually removed some near-synonyms as well as a range of concepts which could not be found in several of the minority-language dictionaries. This did away with many concepts that tend to be expressed by loans from Russian in the minority languages, often covering all aspects of life that are not tied to the traditional cultures. Also, since our concepts were not sufficiently disambiguated for automated cross-language lookup, some pairs of concepts turned out to be practically synonymous. For example, our initial ranking included one concept described by the German glosses *Boot* “boat” and *Kahn* “barge”, and a second concept described by the glosses *Boot* “boat” and *Schiff* “ship”. While the first concept could be used to refer to a smaller vessel than the second one, maintaining a clear separation of such concepts while mapping lexical entries across many gloss languages would be very difficult. Faced with many such examples, we decided to remove most of the concept pairs leading to many duplicate entries in our automated extraction, while keeping some of them in because of our impression that these concepts are more clearly separated in relevant languages than they are in our primary gloss languages Russian, English, or German. One such instance is the separation of the concept of understanding into being able to hear what is said, and grasping the meaning of the message. In a final step, we added some very frequently borrowed concepts, such as days of the week and month names, as simple test cases for loanword detection methods.

The resulting concept list can be separated into 480 nominal concepts which are expressed as nouns in the vast majority of the world’s languages, and 340 verbal concepts. A third category contains 102 qualities which are expressed by adjectives in English and many other languages where this category exists, and by verbs in some others (such as Korean, and to a certain extent Chinese). A final category contains 94 additional concepts of miscellaneous types, such as pronouns, simple adverbs, numbers, and some spatial relations.

Our full list is given in Table 3 in Appendix B, grouped into 36 semantic categories in order to make it easier to get an impression of the overall structure and coverage. The annotations are sometimes needed to distinguish word senses in English (e.g. ‘to show (*let someone see*)’ vs. ‘to show (*be visible*)’), and often in order to select one sense as basic if languages make lexical distinctions within the region of semantic space denoted by the English term. For instance, many languages lexically distinguish the substance one breathes from the space in which birds move, making it necessary to further specify the English equivalent “air”. For some concepts (especially kinship terms), the specification also defines which term is to be mentioned first in our list of equivalents if a more fine-grained distinction is made in the target language, e.g. ‘grandfather (*e.g. father’s father*)’.

The overlap of our 1016-concept list with the 1329-concept IDS list is only 582. In contrast, the only items missing from the WOLD-derived 100-item Leipzig-Jakarta list of stable concepts (Tadmor 2009) are “in” (which is frequently expressed by case in North Eurasian languages), “to grind” (which is not as prominent in the absence of agriculture), and “to suck” (which is difficult to find in Russian dictionaries, presumably for taboo reasons). Of the 207 items contained in the original Swadesh lists (Swadesh 1952, 1955), the starting point of and still a popular choice in lexicostatistics, 184 concepts are also on our list. The missing concepts are predominantly from the cultural sphere (which implies low stability), tool names and agricultural terms, as well as animal and plant names that are not relevant for traditional cultures of Northern Eurasia. These differences highlight that it makes sense not to use one of the existing lists that are tuned towards Indo-European languages, but to create concept lists by means of reproducible methods on a broad sample of languages relevant to the region. While the different sources agree on a core of about 100 items of very stable vocabulary, it is worthwhile to put more effort into deciding on a concept list for a wide-coverage lexical database.

## Data collection and processing

In this section, we describe our data collection and processing workflow. In this description, we will frequently use the terms ‘source language’ and ‘target language’. Unlike in lexicography, the target language will always be the language for which we want to collect the data, and the source languages are the languages in which dictionaries into the target language are available. These names remain the same even if we refer to data from a dictionary that translates target language lemmas back into one of the source languages.

### Design decisions

Many similar large-scale databases (e.g. IDS or WOLD) rely on language experts or native speakers for their data. While this ensures the quality of the result, it is often difficult to get enough experts involved for getting good coverage, and there are only few or no experts or native speakers for many of the smaller languages. Because of this, we decided to initially collect all of the data ourselves in a small team from written sources, and to only ask experts and native speakers later for confirmation and help with missing and unclear data points.

Of course, this means that our initial version is not perfect and still requires corrections and revisions. Also, documentation for many languages is sparse, especially for verbal concepts, and we sometimes had to rely on small individual or old and possibly outdated sources. At times, two sources for the same language employed different orthographies which had to be bridged to maintain compatibility. These orthographies would sometimes not adequately render the language’s phonology, and phonological information was often missing or presented in a variety of different transcription schemes.

As primary data sources, we rely exclusively on published dictionaries on paper or in digital formats, from which information is typically extracted manually by going through all of the relevant entries. We initially experimented with using OCR for pre-processing, but found that OCR models perform very poorly especially on bilingual dictionaries, because they tend to be trained on running text for a single language, and do not know how to handle the very peculiar formatting and mixed-language nature of scanned dictionary pages. Post-processing the output of OCR turned out to be just as time-consuming as typing in the relevant information, especially because the extraction process involves various standardization steps, such as normalizing part-of-speech annotations and domain labels.

All lexical items are extracted in the native orthography whenever possible to preserve the information provided by the sources, and to enable automated integration of comparable data from different sources. A phonetic representation is later inferred automatically from the orthography, or stored in addition where necessary (see Sect. 3.3). In all cases, we took the pragmatic approach of using the form used in our dictionary sources to translate our source language lemmas. Typically no attempt was made to reduce lemmas to stems, or to normalize forms in order to represent underlying representations. Only when different sources used different quotation forms we worked into the grammatical system of the respective language, and looked up or derived the desired form in order to ensure consistent treatment. For instance, in the case of qualities in Korean, we opted for using the present determiner form instead of the non-past indicative used for other verbs.

Compared to projects which are composed out of individual expert contributions, our method has the advantage that we are familiar with all the data, and have a good overview over the current status of our word lists, allowing us as the data managers to confidently implement corrections and additions that would require too much long-term commitment from expert contributors. Because we do not solely rely on external help, we can achieve complete coverage of the desired languages and were able to quickly progress in our initial data collection steps. The vast majority of our entries are filled, and the current version of our database, even though not yet reviewed by experts, is already fully functional for its intended purposes.

### Data collection procedure

**Fig. 2:**
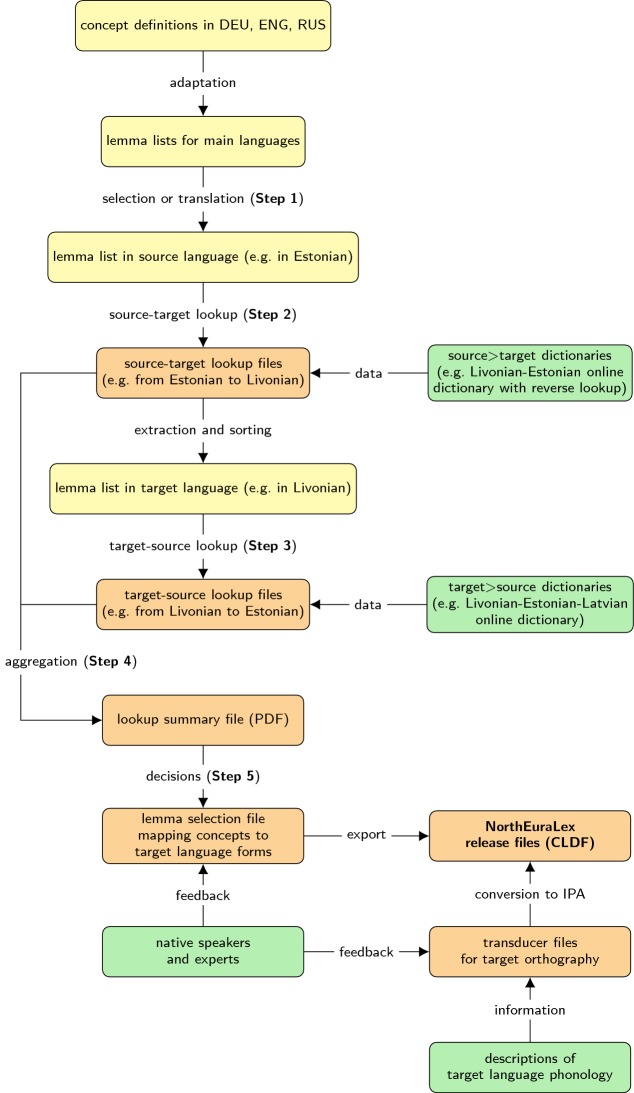
Data collection and processing workflow for NorthEuraLex 0.9. Green nodes represent our sources of information, yellow nodes stand for informal auxiliary files, and orange nodes represent data files in machine-readable standardized formats. (Color figure online)

During the 4 years (2013–2017) that the data collection process lasted, we have developed systematic data collection procedures tuned towards mass processing of dictionary sources by non-experts in the respective target languages. Since to our knowledge, this type of data collection has never before been performed on such a scale, we describe what have shaped up to be our best practices in some detail here. The discussion of our five-step process, which is also outlined in a workflow diagram in Fig. 2, also describes the motivation behind some of our non-obvious design decisions.

When selecting our lexical resources, for target languages where several dictionaries were available, we did not base our decision on the immediate accessibility of the respective source language, but on a preference for work describing the standard language. For many Uralic languages, this implied not to rely on scientific dictionaries in German or English (as seems to be common practice in Western Europe), but on Russian dictionaries of the sometimes very recently established standard languages. Moreover, we always preferred lexical resources where both translation directions were developed independently to resources where one of the two directions is only available as an index or a mechanical inversion of the translation pairs in the other.

(1) *Create lookup lemma list* In order to look up the best equivalents of our 1016 concepts across all the languages of Northern Eurasia, we had to bridge different source languages, for which we needed to develop lookup lemma lists based on our original list. This original list is in German, which became the main language of the database due to the nature of our pre-existing data, and because we found it to be much more efficient and less error-prone to write dictionary entries in the native language of most project members. While all the major source languages we needed to bridge were also target languages in our database, it would not have been optimal to simply re-use the prepared wordlists as lemma lists for lookup, because some most natural choices for a given concept are polysemous, while much more explicit alternative glosses exist. For instance, the concept of shedding tears is most naturally expressed by the verb “to cry” in contemporary English. However, especially in older dictionaries, the translations one finds under “cry” are centered around the concept of shouting, whereas the target concept can much more reliably be looked up under “weep”. A very difficult challenge faced us in the selection of the best Russian equivalents for basic verbs, especially in the domains of movement and manipulation. It is largely unpredictable which of the many corresponding Russian verbs, which lexicalize slight differences in grammatical aspect and other meaning components, is used for this purpose in small dictionaries. In larger dictionaries, the nuances expressed by the different verbs are often quite faithfully, but unnaturally modeled by regular derivational morphology in the target language. Comparing different sources and getting an impression of the most common practices, in most cases we decided to use the perfective forms (often with a disambiguating prefix) as the most useful Russian lookup lemma. Many such considerations were involved in the development of our lookup lemma lists for English, German, and Russian. These three we consider the official gloss languages of the database because together, they provide enough sources to cover the bulk of North Eurasian languages. In addition to these three primary languages, our intention to only use the best available resources for each language created a need for lookup in a surprisingly large number of smaller languages. For these smaller languages, we did not produce independent lookup lemma lists as for the major languages, but took the less ideal decision of relying on the previously collected data for the source language instead. This was our strategy for the following source languages: Norwegian (for the Western Saami languages), Swedish (for some information on South Saami), Finnish (for Inari Saami and Skolt Saami), Estonian and Latvian (for Livonian), Hungarian (for some information on Northern Mansi and Nganasan), French (for Breton), Japanese (for some information on Ainu), and Chinese (for some information on Manchu).

(2) *Lookup in source–target direction* The second step is to look up all the lemmas in a source–target dictionary (e.g. Estonian–Livonian) and digitalize the relevant parts of the target entries for each lemma as faithfully as possible while adapting them to our internal formats. This means that all equivalents are stored in the order in which they appear in the source, annotations given in the source (e.g. abbreviations such as *fig.* for ‘figurative’, and disambiguating information such as prototypical objects for verbs) are extracted, and care is taken to distinguish the separators between different senses of a lemma (frequently a semicolon) from those between alternative translations (frequently a comma). Working with a small team of data collectors coordinated by a single person made it possible to ensure largely consistent and standardized annotations across languages, a prerequisite for the later Step 4 in which all the extracted information is pre-processed automatically. Representation in the original standard orthographies ensures ease of automated retrieval, and compatibility across different resources.

(3) *Lookup in target–source direction* The third step is the reverse lookup stage, where all lemmas in the target language that were collected in the previous step, which can amount to several thousand depending on the size of the dictionary, are looked up in a target–source dictionary (e.g. Livonian–Estonian). The lookup list for this step is produced by automatically inverting the completed lookup list from Step 2, and sorting it alphabetically in the target language for more efficient lookup. Otherwise procedures and formats are exactly the same as in the preceding lookup step, creating mirror lists which model the lexical correspondences from the viewpoint of both languages, often making it possible to resolve possible polysemies. Usage information especially on verbs which can be extracted from example sentences found in good dictionaries, is frequently encoded in additional annotations to further enrich the decision basis.

(4) *Automated aggregation* In the fourth step, the gathered lemmas are mapped back onto the primary concept list. First, the looked-up source language translations are automatically mapped to one of the gloss languages (typically German, the native language of most contributors), which helps to ensure consistency of translations across target languages. The lemmas of the target language are then assigned to the corresponding concepts in a preliminary version of a selection file. To facilitate the work for the human collector, the system already discards some lemmas based on mismatches between their translations in the two lookup steps. However, the entire data is also compiled into a PDF summary file, which lists all possible translations for each concept, even those discarded in the selection file, together with the translations from both lookups and the annotations provided by the dictionaries. This document, containing up to 500 pages of information about the 1016 concepts in our final list, provides the data compiler with a compact view of all the relevant information to efficiently perform the subsequent lemma selection decisions for each language-concept pair.

(5) *Lemma selection* The fifth and final step consists in manually reviewing the pre-generated selection files which store the selection decisions concerning the best equivalent of each concept in the target language. While automated mapping into a gloss language is used for the automated pre-filtering and to create indices bridging different source languages, the decision process itself is always performed on the original data by a data collector with at least a good passive command of the source languages. If multiple translations seem fitting, one will be selected based on disambiguation information provided by the stored dictionary annotations, consistency across multiple dictionaries (if available), and their order in the dictionary entries (assuming that the most widely used word is listed first in the sources). The latter criterion provides another good reason to prefer school dictionaries over scientific ones, because they tend to focus on a single most natural translation, instead of trying to cover all senses, in the worst case in alphabetical order. If the dictionaries themselves do not provide enough information to make a decision, the data collector will consult other sources, such as additional dictionaries, grammars or websites. For additional example sentences, we sometimes relied on the collaborative database Tatoeba (Ho and Simon 2016), and Google phrase searches in the target language often helped us to clarify the contexts in which words are used. Image searches have proved to be particularly useful for nouns, and even the word picked by translation tools such as Google Translate (as unreliable as automated translation generally is) for a gloss language lemma in the context of a sentence are sometimes helpful in deciding on a lemma. If no translation was found in the source dictionaries, the same additional sources are consulted to cover as many concepts as possible. Concepts for which two or more target translations are required because the target language makes more semantic distinctions than the source language (e.g. ‘older brother’ vs. ‘younger brother’) or where the available resources are not sufficient to make a decision for one term, multiple translations can also be given. These are only used sparingly, however, and one of our rules is to never use more than three translations. Each selection decision is annotated by one of four status values describing our level of certainty. The possible statuses are “Questionable” for concept-language pairs for which no information or only suspiciously-looking data from unreliable sources like Google Translate was available, “Review” for decisions that we are uncertain of, typically due to ambiguous sources, and which would need to be reviewed by an expert like a native speaker or a linguist specializing in the language. “Validate” is the status of decisions where the sources seemed quite clear and we have no evidence contradicting our choice. On decisions with this status (which comprise almost 90% of the released database), we would still like to get confirmation by experts, but we do not consider these to be a very high priority. Finally, “Validated” is the status of selection decisions that have already been checked and confirmed by experts or native speakers. To facilitate further review, all data collected in the individual steps (source–target lookup, target–source lookup, selection) is retained and archived. In addition to their key role in the generation of lookup reports, separate machine-readable files for each type of information also facilitate the automation of consistency checking. Also, these files help to remove the need to go back to the primary sources on paper during subsequent steps of the revision process.

### Deriving phonetic representations

For computational approaches that do not start on the level of cognacy decisions, the main problem of many existing lexical databases is that they primarily focus on cognacy judgments, and that little effort is put into standardized and detailed phonetic transcriptions. The differences between the transcriptions employed by the different databases also makes it quite difficult to combine their data in order to derive larger aggregate databases with better coverage.

The Austronesian Basic Vocabulary Database, for instance, while providing quite uniform transcriptions for languages which do not have an official orthography, only contains the written forms for those languages that do. ASJP consistently uses its own transcription scheme, which however reduces the sounds of the world’s languages to 41 equivalence classes, which do not suffice to adequately transcribe central phonological distinctions in many languages. While in principle, the ASJP encoding defines diacritics which would be able to express many of these distinctions, in practice only the 41 basic symbols are used consistently. IELex provides full IPA transcription, but only for some languages, whereas it relies on a mixture of original orthographies and transliteration for others. The dictionaries contained in the IDS do not even aim to include a uniform phonetic representation across all languages, favoring orthographic forms for most languages instead, and leaving the decision how to represent the words to the expert contributors otherwise.

To retrieve a phonetic representation of our lexical data, we developed a simple transcription system that can automatically transcribe orthographic input to IPA in Unicode with the help of language-specific conversion rules. While phonetic transcriptions for individual words are hard to come by especially for smaller and less well-documented languages, most grammars include at least an overview of the phonology, providing us with information on how words in the language are pronounced.

An obvious challenge for such an approach is that its only source of information from which everything needs to be derived are the dictionary forms. This reliance on written forms causes problems whenever the standard orthography does not fully represent pronunciation. Examples include the non-phonemic weakly voiced vowels which are not represented in the orthographies of Tundra Nenets and Skolt Saami, palatalization in the nominative case of some Estonian nouns (caused by an elided front vowel which is only visible in other case forms), and epenthetic vowels which split up consonant clusters in Armenian and other languages. While substantial effort was put into predicting and implementing these phenomena whenever we became aware of them, in others we opted to reduce complexity by aiming for a phonemic notation that more closely corresponds to the orthography. Since many of these distinctions are not of central importance to historical linguistics, we decided that even a transcription which does not fully cover these phenomena was good enough for a first release. While some of the resulting transcriptions have a hybrid status somewhere between the phonetic and phonemic levels, the level of detail usually suffices to accurately represent distinctions which are relevant e.g. for sound correspondences. Pending the expert feedback leading to even better transcriptions, the automated transcriptions generally provide a good approximation of each word’s pronunciation which at least makes uniform application of algorithms across languages feasible.

The main advantage of an automatic transcription system, even if it does not always yield a perfect output, is that expert feedback on the results does not have to be applied manually to each affected word, but can usually be integrated as a new or modified rule to systematically adjust faulty transcriptions. Also, different contributors who manually write IPA transcriptions for words in a language will unavoidably disagree on some details, whereas using an automated system ensures consistency across an entire wordlist. If there are exceptions to the language’s general pronunciation rules, a very common phenomenon in loanwords, our infrastructure allows to override the automatic conversion by specifying the transcription directly in the word list. These mechanisms make our design much more flexible than the approach taken by other databases where phonetic transcriptions are considered primary data which are maintained separately. The effort required for manual revisions makes it much more unlikely that existing transcriptions will be revised and adapted if e.g. it turns out the same sound was represented by different experts in an incompatible way. Our impression is that these difficulties are the main reason why databases of expert contributions like IDS or WOLD do not contain uniform phonetic representations.

In our system, the typical transcriptor for a language is defined by one or more plain text files containing lists of simple rewrite rules. In order to facilitate human editing of these rules, all input is first converted to X-SAMPA (Wells 1995), and these X-SAMPA transcriptions are then transformed into IPA in a second step. The rules can have the form sch$\rightarrow$S to model simple letter-to-sound correspondences. To represent more complex phonological processes, it is possible to specify symbol classes such as frontVowel = [e i ä ö ü] and backVowel = [a o u]. These classes can then be referenced in a rule to systematically change symbols in certain environments, as in properly converting German ch into the *ich* and *ach* sound: [frontVowel]ch$\rightarrow$[.]C and [backVowel]ch$\rightarrow$[.]X, where [.] represents the class on the left side. There can be arbitrarily many classes in a rule and the classes can contain an arbitrary amount of symbols and strings. We found that these two rule types are sufficient to concisely model the grapheme–phoneme correspondences of most languages.

The transcriptor program tries to apply each of these rules in a given file in order and greedily consumes a substring once it has been matched by a rule. Within the same rule file, that string cannot be matched again (e.g. to convert the front and back vowels of the previous example to their X-SAMPA equivalents). However, the output of the application of each rule file serves as the input to the next, so any number of files may be chained together to achieve the desired end result. It is often practical to place each type of phonetic process in a separate file, so that the generation of the final form proceeds in logically separate steps (e.g. Icelandic *öngull*$\rightarrow$öNkudl$\rightarrow$9yNkYdl$\rightarrow$9yNkYtl_0$\rightarrow$ ).

In the future, this simple transcription system is going to be replaced by a finite-state based system, which is an interface between our previously developed transcription rule files and the Helsinki Finite State Toolkit (HFST; Koskenniemi and Yli-Jyrä 2008). It converts the rule files to a series of regular expressions, from which HFST is able to construct the corresponding finite state transducer. Once this transducer has been created, it can be reused to quickly transcribe any input in the given language. This system is faster, especially on longer words and sentences, and could thus also be employed for transcribing whole texts. Also, the underlying transducer works independently of our system and can be distributed on its own. Additionally, HFST offers convenient tools to convert transducers into the internal formats of various other finite state tools.

These HFST-based orthography-to-IPA transducers, and the code for compiling them from our rule files, will be made publicly available as part of an additional publication, which also describes transducer development in more detail. Together with the code, we also plan to release all the transducer definition files for the 103 languages for which we have automated transcription modules from either the orthography or standard transcriptions (e.g. Persian, Pashto, Japanese, Chinese).

**Fig. 3:**
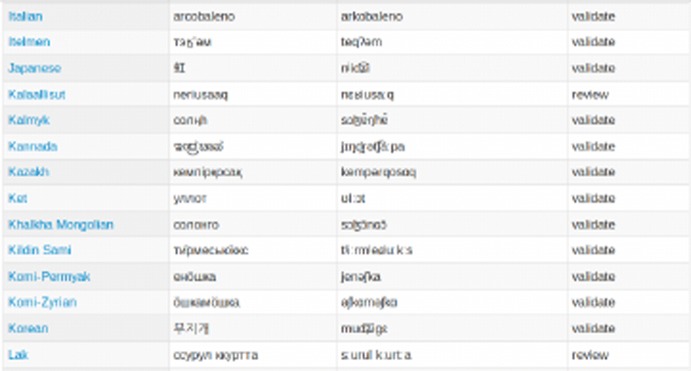
Sample of words for ‘rainbow’ in the web interface

In order to illustrate what the result of the entire workflow looks like in the current database release, we display a sample from the table containing the words for ‘rainbow’ in Fig. 3. In this snippet, we see many different writing systems, one language where we need to generate the IPA out of a standard phonetic transcription (e.g. Japanese), one where some additional phonetic information from the dictionary needed to be modeled (e.g. Kalmyk), and many others where the pronunciation is quite predictable from the orthography, allowing us to automate the mapping using a chain of transducers. The last column contains the mentioned status values for each selection decision, in this case implying that we are quite uncertain about the words in Kalaallisut and Lak, and would prioritize these words when getting into contact with an expert and native speaker, whereas we are reasonably certain already about our choices for all of the other words.

The preliminary release version 0.9 of NorthEuraLex has been available via the project webpage^1^ for inspection and download since July 2017, and we are officially releasing it with this article. The web interface builds on the CLLD framework by Forkel et al. (2018a), which Pavel Sofroniev adopted for the purposes of this project. The database is licensed under a CC-BY-SA license, allowing anyone to use or extend it however they wish, as long as the original version is attributed to us by citing this article, and any extensions are published under the same terms. This policy goes a long way towards ensuring long-term availability, as evidenced by the fact that it has already been added to the Zenodo online repository in a repackaged form.^2^ This version is in the Cross-Linguistic Data Format (CLDF) as described by Forkel et al. (2018b), a linked data format which is quickly becoming the standard for lexicostatistical databases, and will also be the release format for all future versions of our database. Along with the web interface, the CLDF specification is also best reference for readers seeking to explore the possibilities of using the NorthEuraLex database in their own research.

We cannot cite all of our primary sources in this article, but they are documented both in the web interface (in table format as well as next to the wordlists) and in the sources.tsv file of the CLDF release. The web interface includes a warning that our database does not represent a primary resource for any of the languages concerned, and that users who are interested in the data for a particular language, and intend to use lexical data in a context where some erroneous datapoints would lead to problems, should consult and cite these primary sources instead.

## Evaluation

Due to its unconventional approach of data collection by a small group of non-experts, quality control in our database can be expected to be more difficult than in the expert-contribution model. However, looking at expert-contribution databases like IDS, it becomes clear that the quality of expert contributions, even if there are no obviously wrong entries, can suffer from misunderstandings about the concepts, or the intentions of a lexicostatistical database. For instance, experts have a tendency to provide long lists of all the words that can be used for some concept, instead of focusing on the single most salient one. Also, it can be difficult to convince an expert contributor to adhere to a cross-linguistic standard of representation, because research traditions differ vastly from family to family. These are issues which can be handled much more smoothly in our centralized approach.

On the negative side, our data collection strategy inevitably leads to some erroneous entries if measured against the goal of retrieving the most salient or natural lexeme for each concept. Missing frequency information only aggravates the problems caused by misinterpretation of dictionary entries in some of the less familiar source languages. Better results could be achieved by considering parallel corpora. However, while small corpora for many minority languages exist, and are extremely valuable resources for many linguistic questions, they typically contain only a few thousand sentences with translations into a more widespread language. For reliable lexicographic decisions based on concordancing, each lexeme from a set of alternatives should ideally occur in dozens of example sentences, which presupposes a corpus of millions of sentences. To improve data quality in NorthEuraLex beyond its current state, it will therefore be vital to rely on the assistance of experts and native speakers of the individual languages.

While focusing on the extraction of large amounts of lexical data from written sources, our work group has been getting in contact with experts and native speakers in order to get an impression of the level of quality we achieved so far, and to predict which level of quality we can expect to be achievable in data collected by linguistically informed non-experts. For this article, we systematically analyzed all our data for a sample of six languages (Italian, Lithuanian, Ukrainian, Hungarian, Udmurt, and Japanese) from three different families. After having every wordlist checked by a native speaker, followed by a thorough discussion of each problematic concept to ensure we established the best possible equivalents, we classified each lexical item that ended up being deleted (or had to be added) into one of eight error classes. The least severe type of error, which in our sample only occurred for the Japanese data, is the choice of an uncommon orthographic variant, in this case picking the Kanji representations for some terms which are most commonly written using Katakana. The error category “wrong register” is assigned to terms which are indeed used for the concept in question, but are not part of the literary standard language, usually because they are considered colloquial or outdated. The error label “low frequency” is used for forms which can be used for the concept in question, but are used a lot less frequently than the other equivalents, and are therefore considered suboptimal choices according to our quality standards. The “added form” tag is applied whenever the correction resulted in more equivalents than were in our originally extracted data, indicating that an important equivalent was absent from our data. The “wrong form” tag is used for words with spelling mistakes, which are not very frequent because many typos are fixed due to lookup in both directions, or where we chose a correct lemma or stem, but in a suboptimal shape, e.g. when we picked the intransitive verb for ‘to break’ when a causative equivalent would have been needed. We consider these five types of errors as not very severe, because the datapoints are either not technically wrong, or unproblematic in a database that is mainly used for lexicostatistics. Three additional error categories are used for errors that we would consider more severe. The first one concerns forms that were rejected by our native speaker informants as dialectal, which only occurred for some Italian and Udmurt words in our experiment. The most common type of severe error is labeled “imprecise match”, indicating that while there are some contexts in which the word could be used to express the intended concept, its meaning is either too general or too specialized to qualify as a good equivalent. Finally, the “wrong choice” tag is used for words that were rejected as not fitting the concept in question under any circumstances. The results for these eight error types across our six-language sample are summarized as percentages in Table 1.

**Table 1:** Classification of errors on our six-language evaluation sample

Error type	ITA (%)	UKR (%)	LIT (%)	HUN (%)	UDM (%)	JPN (%)
Correct form	91.9	92.0	84.7	93.8	83.7	91.8
Orthographic variant	0.0	0.0	0.0	0.0	0.0	1.4
Wrong register	0.8	0.5	1.6	0.2	3.1	0.5
Low frequency	0.7	0.8	1.5	0.4	2.4	0.1
Added form	1.9	1.8	2.1	1.4	0.7	2.5
Wrong form	0.4	0.4	1.1	0.4	1.4	0.3
Dialectal	0.3	0.0	0.0	0.0	0.8	0.0
Imprecise match	2.4	2.9	5.3	2.8	4.4	1.7
Wrong choice	1.6	1.6	3.6	1.0	3.5	1.7

Except for the error types which only occurred in certain languages (orthographic variants and dialectal forms), the figures show similar tendencies across all types. This makes it possible to limit the discussion to the most severe error categories. In those national languages for which high-quality dictionaries were available (Italian, Ukrainian, Hungarian, Japanese), only between 1.0 and 1.7% of entries were considered clearly wrong by native speakers, which we consider a good sign. Wrong choices were typically caused by polysemies of the gloss languages which the dictionaries did not sufficiently disambiguate, or in some cases by misunderstandings in interpreting the available information. The number of suboptimal choices due to imprecise matching typically stay below three percent. The much worse figures for Lithuanian and Udmurt are explainable by differences in source quality, and a lack of readily available example sentences for validating our choices. For a typical minority language, we would expect the error rate to be somewhere in the middle between these values, with Siberian languages (due to the scarcity of resources) and Caucasian languages (due to our lack of grammatical knowledge) being the most problematic in the current version.

Concerning coverage, version 0.9 contains data for 97.8% of all language-concept pairs. For 89.6% of these entries we are quite confident that no changes will be necessary, although we would still like to validate all of the data at some point, while the other 8.2% will be prioritized for review by native speakers or experts when preparing future releases. Among our language sample, only the dataset for Udmurt, one of the larger Uralic languages of Russia, contained lexical gaps due to lack of dictionary coverage. An interesting observation was that all these gaps turned out to be difficult to fill even for a native speaker, indicating that words not listed in the dictionaries will not be very prominent in the target languages. We expect that this situation, in which older dictionaries contain more lexical information than a native speaker will actively remember, will frequently be the case in the typical bilingual situation with a very dominant state language, especially for the many moribund languages which have ceased to function as media of everyday communication.

It is also interesting to observe how the errors are distributed across concepts. If we classify concepts by the worst errors which occurred in any of our six languages, we find that the worst three error categories only ever occur in 316 of our concepts, whereas we would expect between 348 and 375 concepts to be affected by at least one of these errors if our 447 errors of this severity were completely randomly distributed across the 1016 concepts (95% CI based on 1000 simulation runs). In contrast, the data for 511 concepts was found to be without errors across our sample of six languages, whereas we would only have expected between 435 and 472 such concepts if the total of 819 errors of all types were randomly distributed. This indicates that the errors are clustering in a set of concepts which are particulary difficult to reliably extract from dictionaries. We conclude that if needed, a higher-quality subset could be extracted by discarding the data for the most problematic concepts. Based on what we know so far, the most obvious candidates for this would be the concepts with the highest number of languages for which problems were found. The maximum of four out of the six sampled languages was reached by five concepts: the two nominal concepts ‘thigh’ (due to the polysemy of both German *Schenkel* and Russian *bedro*) and ‘business (*commercial activity*)’ (due to diverging attitudes and unwanted connotations connected with loans from English *business*), and the three verbal concepts ‘to turn out (*result, end up*)’, ‘to rise (*e.g. water level*)’ and ‘to sting (*e.g. with a needle*)’, which are all difficult to look up due to a lack of relevant examples in dictionaries.

## Future development

Within our group, the database has already been used for a wide range of applications in computational historical linguistics. The part which is already cognacy-annotated provides us with a large benchmark for automated sound correspondence and cognacy detection. The development of new methods for the detection of contact events on the language level (Dellert 2017) have been another focus of our work with the database. Colexifications extracted from the lexical data have also proved worthwhile in a graph-based computational model of semantic change (Münch and Dellert 2015).

A second project phase, which has started in October 2018, is going to bring NorthEuraLex towards the next release version 1.0. For this version, we have started to collect cognacy and loanword information from etymological dictionaries, with the long-term goal of providing these annotations across the database. At the same time, expansion by 96 additional languages (many ancient languages such as Ancient Greek, Sanskrit, and Hittite, plus additional smaller Indo-European, Turkic, Mongolic, and Tungusic languages) is under way, which will allow version 1.0 to reach almost twice the coverage of the first release version which we have described in this article. These expansion measures will be complemented by continued systematic efforts to improve on the quality of the database with the help of native speakers and experts.

Many of the infrastructure tools we developed for this project could be of value to similar projects. Currently the tools are under intensive continual development due to the ongoing second project phase, but we are already distributing parts of our code to other database projects on request. After the release of version 1.0, which is currently scheduled for some time in late 2020, work on the database will switch to an open development paradigm for the further refinement stages. This includes plans to release the source code for all of our development and data management tools under an open license, and to continue further development in an openly accessible repository in order to facilitate external contributions, and to make development versions between scheduled releases available.

In a more long-term perspective for further expansions, we are planning to add morphological information to all entries in a machine-assisted way, much as we have been doing it for the transcriptions. For this, we will build on our prototype implementation of a system which extracts stem-like segments from the dictionary forms based on information content (Dellert 2018). Since some at least rudimentary morphological analysis tools even for quite a few minority languages in our area do already exist, we will employ these tools whenever possible. We are also evaluating the potential of further improvements to our phonetic representations with the help of audio recordings. Such recordings exist even for many minority languages of our target area, which would help to resolve some questions, especially in the realm of phonotactics, that we could not answer based on the available literature describing the languages’ sound systems.

## Conclusion

With this article, we officially release NorthEuraLex, a new wide-coverage lexical database which aims to cover a substantial core vocabulary across all languages of Northern Eurasia. The database differs from similar endeavours by initially not building on expert contributions of wordlists for individual languages, but a systematic process for extracting the necessary lexical data from existing resources, and then only relying on expert and native speaker knowledge for continual revision and improvement. This model has the disadvantage of a higher error rate in early versions, but makes maintenance and coordination of future revisions and improvements considerably easier. This is of course only possible because in the relevant geographical region, the lexicon of most languages is well-documented by published resources, making it possible to complete much preparatory work before needing to switch to unpublished field notes or new fieldwork.

The first release version 0.9 already provides more than 100,000 words in a unified IPA encoding, covering 107 languages from 21 families. To our knowledge, it is currently the only wide-coverage IPA-encoded database spanning an entire geographical region instead of focusing on a single language family. Coverage of the target area is still far from complete, which is why NorthEuraLex is going to grow to almost twice its current size in the next release version, and pending further funding, we are planning to expand it further in order to achieve full coverage of our inventory of 350 sufficiently well-documented languages across the entire linguistic area within the next 5 years.

## Appendix A: List of languages

See Table 2.

**Table 2:** List of languages in NorthEuraLex 0.9

Family	Subfamily	Languages (with ISO 639-3 codes)
Abkhaz-Adyge	Abkhaz-Abaza	Abkhaz (abk)
	Circassian	Adyghe (ady)
Afro-Asiatic	Semitic	Standard Arabic (arb), Modern Hebrew (heb)
Ainu	Hokkaido-Kuril Ainu	Hokkaido Ainu (ain)
Basque	Basque	Basque (eus)
Burushaski	Burushaski	Burushaski (bsk)
Chukotko-Kamchatkan	Chukotian	Chukchi (ckt)
	Itelmen	Itelmen (itl)
Dravidian	South Dravidian	Kannada (kan), Malayalam (mal), Tamil (tam), Telugu (tel)
Eskimo-Aleut	Aleut	Aleut (ale)
	Eskimo	Central Siberian Yupik (ess), Kalaallisut (kal)
Indo-European	Albanian	Standard Albanian (sqi)
	Armenic	Armenian (hye)
	Baltic	Latvian (lav), Lithuanian (lit)
	Celtic	Breton (bre), Irish (gle), Welsh (cym)
	Germanic	Danish (dan), Dutch (nld), English (eng),
		German (deu), Icelandic (isl),
		Norwegian Bokmål (nob), Swedish (swe)
	Graeco-Phrygian	Modern Greek (ell)
	Indo-Aryan	Bengali (ben), Hindi (hin)
	Iranian	Northern Kurdish (kmr), Pashto (pbu),
		Ossetian (oss), Western Farsi (pes)
	Italic	Catalan (cat), French (fra),
		Italian (ita), Latin (lat), Portuguese (por),
		Romanian (ron), Spanish (spa)
	Slavic	Belarusian (bel), Bulgarian (bul),
		Croatian (hrv), Czech (ces), Polish (pol), Russian (rus), Slovak (slk), Slovene (slv), Ukrainian (ukr)
Japonic	Japanesic	Japanese (jpn)
Kartvelian	Georgian-Zan	Georgian (kat)
Koreanic	Korean	Korean (kor)
Mongolic	Eastern Mongolic	Buryat (bxr), Khalkha (khk), Kalmyk (xal)
Nakh-Daghestanian	Daghestanian	Avar (ava), Dargwa (dar), Lak (lbe),
		Lezgian (lez), Tsez (ddo)
	Nakh	Chechen (che)
Nivkh	Nivkh	Nivkh (niv)
Sino-Tibetan	Sinitic	Mandarin Chinese (cmn)
Tungusic	Central Tungusic	Nanai (gld)
	Northern Tungusic	Evenki (evn)
	Manchu-Jurchen	Manchu (mnc)
Turkic	Bolgar	Chuvash (chv)
	Kipchak	Bashkir (bak), Kazakh (kaz), Tatar (tat)
	North Siberian Turkic	Sakha (sah)
	Oghuz	North Azerbaijani (azj), Turkish (tur)
	Turkestan Turkic	Southern Uzbek (uzs)
Uralic	Finnic	Estonian (ekk), Finnish (fin), Livonian (liv), North Karelian (krl), Olonets Karelian (olo), Veps (vep)
	Hungarian	Hungarian (hun)
	Khantyic	Northern Khanty (kca)
	Mansic	Northern Mansi (mns)
	Mari	Hill Mari (mrj), Meadow Mari (mhr)
	Mordvin	Erzya (myv), Moksha (mdf)
	Permian	Komi-Permyak (koi), Komi-Zyrian (kpv),
		Udmurt (udm)
	Saami	Inari Saami (smn), Kildin Saami (sjd),
		Lule Saami (smj), Northern Saami (sme),
		Skolt Saami (sms), Southern Saami (sma)
	Samoyedic	Forest Enets (enf), Nganasan (nio),
		Northern Selkup (sel), Tundra Nenets (yrk)
Yeniseian	Northern Yeniseian	Ket (ket)
Yukaghir	Kolymic	Southern Yukaghir (yux)
	Northern Yukaghir	Northern Yukaghir (ykg)

## Appendix B: List of concepts

See Table 3.

**Table 3:** List of concepts in NorthEuraLex 0.9, split into categories

Domain	Concepts
Animals	animal, ant, bear, bird, bull, butterfly, cat, chicken, claw (*e.g. of a bird*), cock (*male chicken*), cow, crane, crow, cuckoo, dog, duck, eagle, egg (*e.g. of a bird*), elk, feather, fish, flock (*e.g. of sheep*), fly (*insect*), fox, fur, gnat, goose, hare, horn, horse, lair (*e.g. of a fox*), louse, mouse, nest (*e.g. of a bird*), owl, paw (*e.g. of a cat*), perch, pig, pike, sheep, snake, spider, squirrel, swan, swarm (*e.g. of birds*), tail (*e.g. of a dog*), track (*e.g. of an animal*), wing, wolf, worm, to bark (*of dog*), to howl (*e.g. of wolf*)
Artifacts	broom, bag (*container for carrying*), bucket, bundle (*group of objects tied together*), cauldron, cover/lid (*of a container*), cup, dishes (*dishware, crockery*), doll, figure (*e.g. representing a god*), fork (*for eating*), handle (*part of an object which is held*), hook, knife, leash (*e.g. dog leash*) lock, nail (*spike-shaped metal fastener*), net (*mesh of string*), picture, pot (*vessel for cooking*), pouch, sack, shovel, spade, spoon
Calendar	April, August, December, February, Friday, January, July, June, March, May, Monday, November, October, Saturday, September, Sunday Thursday, Tuesday, Wednesday
Classification	angle (*internal, e.g. of a room*), area (*extent of surface, region*), circle (*geometric figure*), corner (*external angle*), count (*quantity counted*), cross (*geometric figure*), distance (*between two points*), edge (*sharp terminating border*), end (*of a long object*), fringe (*peripheral part*), gap (*between objects*), half (*of a quantity*), heap, hole (*in an object*), item (*physical object*), line (*geometric figure*), matter (*affair*), middle (*central part of something*), part (*fraction of a whole*), pattern (*regular repeated elements*), piece (*separable part of a larger whole*), row (*line of objects*), side (*of an object*), sort (*type*), space (*available space, e.g. in a closet*), thing (*distinct entity*), tip (*of a pointy object*), to alter (*make different*)
Clothing	belt (*clothing*), blanket (*for sleeping*), boot (*heavy shoe covering part of the leg*), button (*fastener on clothes*), cap (*head covering*), clothes, cloth (*woven fabric*), collar (*e.g. on a coat*), knot (*looping of string*), leather, needle (*for sewing*), paint (*substance for colouring*), pillow (*for sleeping*), ribbon (*strip of cloth*), ring (*jewellery*), scarf (*light, worn around the shoulders*), shirt, shoe, sleeve, string (*made from twisted threads*), thread, to dye (*e.g. hair*), to get dressed, to knit (*e.g. a jacket*), to put on (*e.g. one’s coat*), to sew, to take off (*e.g. one’s coat*), trousers, wool
Crafts	ash (*solid remains of a fire*), board (*long, wide and thin piece of wood*), clay (*ductile material*), coal (*combustible substance*), glass (*transparent substance*), gold (*metal*), iron (*metal*), noose (*e.g. on a rope*), pipe (*hollow conduit, tube*), pole (*long and slender piece of wood*), sand (*material*), silver (*metal*), slab (*flat piece of solid material*), staff (*long straight stick*), stick (*piece of wood used as a tool*), strap (*e.g. strip of leather*), support/rest (*something which keeps upright*), wood (*material*), to build (*e.g. a house*), to make (*create, bring about*), to produce (*manufacture*), to repair/mend (*e.g. a vehicle*)
Emotions	dear (*beloved*), desire (*to have something*), grief (*sorrow, sadness*), happiness, joy (*emotion*), laughter, sad (*emotion*), wish (*longing*), to be afraid of (*fear*), to be afraid (*be frightened*), to be annoyed (*be irritated*), to cry (*weep, shed tears*), to flee (*run away*), to grumble (*complain in a surly manner*), to laugh, to like (*favor, be in favor of*), to love (*have a strong affection for*), to move (*arouse the feelings of*)
Housing	bed, box, bridge, chair, cradle, door, fence, floor (*supporting surface of a room*), gate, home (*one’s own dwelling place*), house (*building*), ladder, pasture, path (*trail used by pedestrians*), road (*way for travelling by vehicle*), roof, shelf, stove (*heater*), table, town, village, way (*connection between places*), well (*source of water*), window, to dwell
Human body	arm, back, beard (*generic*), belly, blind, blood (*fluid*), body (*of a living organism*), bone, bosom (*female breast*), brain, breast (*e.g. of a man*), breath (*breathing*), cheek, chin, deaf, dream (*while sleeping*), ear, elbow, eye, face, fat (*person*), fever, finger, fingernail, foot, forehead, hair (*on head*), hand, head, health, heart, heel, hunger (*condition*), hungry, ill, illness, jaw, knee, leg, lip, liver, medicine (*drug*), moustache, mouth, nail, naked, nape (*back side of neck*), navel, neck (*from outside*), nose, pain, palm, pretty, shoulder, sinew, skin, sleep (*condition*), slim, stomach, strength (*being strong*), strong (*tough*), tear (*drop of fluid*), thigh, throat (*from inside*), toe, tongue, tooth (*e.g. incisor*), vein, weak (*feeble*), wound, to ache (*e.g. of head*), to be ill, to blow (*using the mouth*), to breathe, to cough, to drink, to eat, to fall asleep, to fall ill, to get tired, to get up (*rise from one’s bed*), to groan (*e.g. in pain*), to recover (*from an illness*), to relax (*recreate*), to shout, to sleep, to swallow, to tremble (*e.g. with fear*), to wake up, to whistle
Hygiene	comb (*implement for grooming*), filth (*that which soils or defiles*), mirror, to clean (*e.g. using a brush*), to comb, to decorate (*e.g. a house*), to have a bath (*e.g. in a river*), to rinse (*e.g. clothes, dishes*), to sweep (*e.g. a room*), to wash (*e.g. clothes*), to wash *oneself*, towel (*used for wiping*), to wipe
Kinship	aunt (*e.g. father’s sister*), boy (*male child*), brother (*e.g. elder brother*), child (*e.g. 10 years old*), daughter, family (*group of close relatives living together*), father, girl (*female child*), grandfather (*e.g. father’s father*), grandmother (*e.g. father’s mother*), human (*human being*), husband, man, mother, parents, sister (*e.g. elder sister*), son, uncle (*e.g. father’s brother*), wife, woman
Language	call (*appeal*), fairy tale, language, message, name (*of a person*), news, puzzle (*riddle*), sign (*object bearing a message*), song, speech (*long oral message*), story (*oral narration*), talk (*conversation*), voice (*sounds uttered by vocal cords*), word (*unit of language*), to ask (*question*), to brag, to call (*someone to come*), to chat (*have a conversation*), to convey (*e.g. a message*), to name (*give a name to*), to request, to say (*e.g. utter*), to speak (*produce utterances*), to talk (*exchange information*), to tell, to translate (*e.g. text*)
Manipulation	force (*which is applied to produce an effect*), to add, to bend (*e.g. a pipe*), to bite (*e.g. a child, of a dog*), to bow (*e.g. the arm*), to break (*e.g. a plate*), to bring, to burn (*e.g. paper, wood*), to carry (*e.g. a box*), to catch (*e.g. a ball*), to choose (*between options*), to close (*e.g. a window*), to connect (*join, e.g. using strings*), to cover (*e.g. a boat using a canvas*), to cut off (*e.g. a piece of cake*), to cut (*e.g. paper*), to damage (*e.g. a house, a vehicle*), to destroy (*e.g. a village*), to dig (*in earth*), to divide (*split into parts*), to drag (*e.g. the trunk of a tree*), to drop (*let fall from one’s hands*), to fill (*e.g. a glass with water*), to get (*obtain, e.g. an important item*), to glue (*e.g. a poster to a wall*), to grab (*e.g. someone’s arm*), to hang up (*e.g. a picture*), to hide, to hit (*on purpose, e.g. a table*), to hold (*in one’s hands*), to jog (*e.g. on a door*), to keep (*e.g. food, money*), to knock (*e.g. on a wall*), to lay (*e.g. money onto a table*), to leave (*e.g. a coat at home*), to lick (*e.g. a wound*), to lift (*e.g. a box*), to light (*e.g. a candle*), to lose (*e.g. a key*), to open (*e.g. a window*), to pick up (*from the floor*), to place (*cause someone to sit*), to pour (*e.g. water into a glass*), to preserve, to press (*exert weight or force against*), to pull (*e.g. a rope*), to push (*shove, e.g. a box*), to put (*in upright position, e.g. a glass*), to raise (*e.g. one’s hand*), to rub (*e.g. glass with a rag*), to scrape (*e.g. a plate using a knife*), to seize (*grab, e.g. a knife*), to select (*pick one option*), to send (*e.g. a letter*), to shake (*e.g. a bottle*), to sharpen (*e.g. a knife*), to spin (*e.g. a skewer*), to spread (*e.g. fat on a surface*), to stick (*e.g. a twig into a hole*), to sweep (*e.g. leaves*), to swing (*e.g. a flag, cloth*), to take, to tear off (*e.g. a doll’s arm*), to tear (*e.g. a cloth*), to throw (*e.g. a ball*), to tie (*e.g. using a rope*), to touch (*make physical contact with*), to turn around (*e.g. palm*), to turn over (*e.g. pancakes, pieces of meat*), to wrap (*e.g. a fish in paper*)
Mathematics	a hundred, a thousand, amount (*measurable quantity of a material*), eight, eighty, eleven, fifty, first, five, forty, four, height, last (*in a row*), length, nine, ninety, one, second, seven, seventy, six, sixty, size (*dimensions of an object*), ten, third, thirty, three, twelve, twenty, two, weight (*mass as measured*), to calculate (*reckon*), to count (*determine the number of*), to decrease (*of quantity*), to increase (*of quantity*), to measure (*e.g. ascertain the height of*)
Modality	can (*be able to*), to do (*e.g. “what are you doing?”*), to finish (*e.g. a piece of work*), to start (*initiate, e.g. a quarrel*), to stop (*stop the current activity*), to succeed (*be successful*), to turn out (*result, end up*), to want (*desire*)
Movement	to arrive (*reach one’s destination*), to budge (*change posture*), to climb (*of human*), to come back (*return to a place*), to come (*move here*), to creep (*of human*), to dash (*e.g. of a vehicle*), to depart (*set out on a journey*), to dive (*of human*), to drop (*e.g. water level*), to enter (*e.g. a building, a room*), to escape (*get away, e.g. from captivity*), to fall (*swiftly move downwards*), to flow (*e.g. a river*), to fly (*e.g. of bird*), to go (*move through space*), to go away (*leave a place*), to go in (*go into an enclosed space*), to hurry (*do things quickly*), to jump (*of human*), to move (*change place*), to revolve (*e.g. of a wheel*), to rise (*e.g. water level*), to run (*of human*), to rush (*move in haste*), to seesaw (*use a seesaw*), to sink (*e.g. stone in water*), to sit down (*assume a sitting position*), to stand up (*e.g. from a chair*), to stay (*remain in a place*), to step (*move the foot in walking*), to stop (*cease moving*), to stream (*to flow in a continuous manner*), to sway (*move backward and forward*), to swim (*of human*), to swing (*repeatedly move from side to side*), to take a walk, to tumble (*fall end over end*), to walk (*move on the feet*)
Nature	air (*where birds fly*), bay (*small coastal inlet*), brook (*small river*), cave (*in a mountain*), chill (*low temperature*), cloud (*bright*), coast (*seashore*), current (*of a river*), dirt (*on objects*), drop (*e.g. water*), dust (*settled*), earth (*substance*), elevation (*of the ground*), fire (*flames*), foam (*on liquid*), fog, forest, frost (*temperature below freezing point*), ground (*soil, earth*), heat (*high temperature*), hill (*possibly wooded*), hoarfrost, ice, island, lake, land (*as opposed to sea*), light (*from a natural source*), meadow (*land covered with grass*), moon (*celestial body*), moor (*wasteland covered by heath*), mountain (*woodless*), pit (*hole in the ground*), rainbow, rain (*falling*), river (*larger river*), sea, shadow (*shady place*), shore (*of a lake*), sky (*visible*), slope (*of a mountain*), smoke (*from a fire*), snow (*on the ground*), source (*of a river*), spark (*from a fire*), star (*in the night sky*), stone (*substance*), summit (*of a mountain*), sun (*celestial body*), swamp (*area of impassable wet land*), thunder (*during a thunderstorm*), water (*cold water*), wave (*on water*), weather, wind (*outside*), to blow (*of wind*), to boil (*of water*), to break (*e.g. a bone*), to burn (*e.g. of wood*), to decay (*e.g. of wood*), to dry (*e.g. of clothes*), to freeze (*to become solid due to low temperature*), to melt (*e.g. of ice*), to rain (*e.g. “it is raining”*), to rise (*of the sun*), to rot (*e.g. of meat*), to set (*of the sun*), to shine (*of the sun*), to smoke (*give off smoke*), to snap (*e.g. of a thread*), to thaw (*e.g. of snow*), to thunder (*e.g. “it thundered”*)
Negative interaction	damage (*material harm, detriment*), fault (*blame, responsibility*), lie (*falsehood*), misfortune (*undesirable condition*), mistake (*wrong action or decision*), to annoy, to beat (*someone*), to bother, to brawl, to cheat (*e.g. someone of money*), to conceal (*e.g. a fact*), to disturb, to hinder, to kick (*someone, a dog*), to kill, to leave (*someone*), to separate (*part ways*), to shove (*someone*), to steal, to sting (*e.g. with a needle*)
Personality	clever (*intelligent*), diligent, evil (*malevolent*), gentle (*tender*), lazy, merry, skilful, stingy, stupid
Place	above, ahead, back (*to the start*), backward, behind, below, between, down, everywhere, far (*distant*), forward, hence, here, hither, in front of, left (*e.g. left leg*), near (*close*), next to, place (*location, position*), right (*e.g. right leg*), there, thither, through (*e.g. a window, a hole*), under, up, to hang (*e.g. “the coat is hanging”*), to lie (*of a person*), to sit (*of a person*), to stand (*of a person*)
Plants	apple, bark, berry (*generic term*), birch, fir, flower, grain (*single grain*), grass (*ground cover*), hay, leaf, limb (*of a tree*), mushroom (*generic term*), onion (*edible*), peel (*e.g. of an apple*), pine, root, seed (*fertilized grain*), tree, trunk (*of a tree*), twig, willow, to grow, to ripen
Politics	border (*between states*), country (*set region of land*), king, power (*ability to control*), state (*sovereign polity*), to rule (*e.g. country*)
Positive interaction	to assemble (*e.g. people in a place*), to bring up (*raise, e.g. a child*), to cover (*e.g. a child with a blanket*), to dance, to give (*an object, e.g. a fruit*), to give (*as a present, e.g. a book*), to guide (*e.g. to one’s destination*), to hand (*e.g. salt at table*), to instruct (*e.g. children*), to invite (*to one’s place*), to kiss, to lead, to let (*cause someone to*), to meet, to play (*e.g. of children*), to praise, to promise, to receive (*be given*), to rescue (*someone*), to rock (*a child*), to show (*let someone see*), to sing, to teach (*e.g. to swim, to sew*), to unite (*form into a group*), to urge, to visit (*e.g. a friend*), to wake, to wish (*e.g. luck*)
Pronouns	this, that, everything, I, thou, he, we, they, you (*plural*)
Qualities	beautiful (*e.g. flower*), big (*e.g. rock*), blunt (*e.g. knife*), clean, closed (*e.g. door*), cold, cool, damp, delicate/fine (*e.g. yarn, web*), dense (*e.g. hair, forest*), dirty, dry, empty (*container*), firm/solid (*e.g. wood, wall*), flat (*object*), fresh (*e.g. air*), full (*container*), hard (*e.g. shell*), heavy (*of weight*), hot (*e.g. fire*), little (*e.g. rock*), long (*object*), narrow (*e.g. river, bridge*), open (*e.g. door*), pointed (*e.g. needle*), powerful (*forceful*), raw (*uncooked*), ripe (*e.g. fruit*), round (*circular*), sharp (*e.g. knife*), short (*object*), smooth (*surface*), soft (*e.g. cushion*), thick (*object*), thin (*object*), warm, wet, wide (*e.g. river, bridge*)
Questions	how, how much, what, where, where to, who, why
Religion	church, death, god, grave, life (*state of being alive*), living, sin, spirit (*inner energy of a being*), world (*universe*), to be born (*of human*), to die (*of human*), to live (*be alive*), to perish (*die violently*)
Senses	bitter (*taste*), black, blue, bright (*full of light*), calm (*absence of noise and disturbances*), colourful (*multicolored*), dark (*devoid of light*), delicious (*tasty*), flavour (*of something*), green, grey, noise (*unwanted loud sounds*), odour (*of something*), red, sound (*sensation perceived by the ear*), sour (*e.g. lemon*), sweet (*taste*), tone (*sound of a specific pitch*), white, yellow, to appear (*e.g. light, ghost*), to be cold (*feel the sensation of coldness*), to be noisy (*e.g. children*), to chime (*e.g. of bell*), to disappear (*e.g. light, ghost*), to feel (*sense*), to hear (*perceive with the ears*), to hear (*receive information about*), to listen (*pay attention to speech*), to look at (*turn the eyes toward*), to ring (*e.g. doorbell, phone*), to roar (*e.g. storm, sea*), to rustle (*e.g. of leaves in breeze*), to seem (*appear, be perceived as*), to see (*perceive with the eyes*), to shine (*e.g. of metal, shiny surface*), to show (*be visible*), to smell (*sense the smell of*), to sound (*e.g. of instrument*), to sparkle/twinkle (*e.g. of star, little light*), to taste (*sample the flavor of*), to tinkle (*e.g. glass*), to watch (*view for a period of time, e.g. movie*)
Social relations	boss (*supervisor*), companion (*with whom one spends time*), company (*companionship*), doctor (*physician*), friend (*person whose company one enjoys*), game (*playful activity*), gift, guest, help (*assistance*), master (*expert tradesman*), nation (*community defined by common culture*), people (*many persons*), teacher, worker, work (*employment*), to work
Subsistence	bread, butter, corn (*cereal plant grown for its grain*), dish (*type of prepared food*), fat (*refined substance*), food (*consumable substance*), honey, meal (*process of food intake*), meat, milk, mush, oil (*liquid vegetable fat*), salt, slice (*e.g. of bread*), soup, tea, trap (*e.g. mouse trap*), to bake (*e.g. bread, a cake*), to boil (*e.g. vegetables*), to catch (*an animal*), to chop (*e.g. kale*), to cook (*e.g. a meal*), to drive (*cattle*), to feed (*an animal*), to fish (*catch fish*), to fry (*e.g. meat*), to gather (*e.g. mushrooms, berries*), to herd (*cattle*), to hunt, to milk (*a cow*), to pick (*e.g. an apple*), to stir (*e.g. soup*), to tie up (*an animal*), to water (*e.g. flowers*)
Thinking	a little, alone, and, at once (*in one go*), bad (*e.g. tool*), because of, correct, different, familiar, famous, foreign, good (*e.g. tool*), hardly, in vain, meaning (*denotation*), memory (*human ability to record information*), mind (*ability for rational thought*), nasty/dire, not, only (*e.g. only one cow*), or, other, reason (*motive, rationale*), so, thought, together, true (*truthful*), truth, very, to await (*wait for someone*), to believe, to deceive (*create the wrong impression*), to endure (*e.g. pain*), to find (*locate something searched for*), to forget (*lose remembrance of*), to grasp (*understand the meaning of*), to improve (*make better*), to know (*be aware of*), to learn (*e.g. to sew*), to look for (*seek, attempt to find*), to notice, to prepare (*make ready, set up*), to recognize, to remember (*recall from one’s memory*), to spoil (*e.g. the mood*), to think, to try (*attempt*), to understand (*acoustically, e.g. a call*), to wait (*delay action until something happens*)
Time	afterwards, again, age (*number of years since birth*), already, always, at first, at that time, autumn, before, day, end (*conclusion*), evening, former (*erstwhile*), instantly, later, long (*a long time*), month, morning, never, new, night, noon, now, often, old (*e.g. house*), old (*person*), once (*a single time*), once (*once upon a time*), sometimes, soon, spring, still, suddenly, summer, then, time (*constant passing of events*), today, tomorrow, week, winter, year, yesterday, young, to become (*turn into*), to begin (*start existing*), to change (*become something different*), to end (*stop existing*), to happen (*occur*), to pass (*of time*)
Trade	benefit (*advantage*), business (*commercial activity*), cheap, expensive (*goods*), money, poor (*needy*), precious, price, rich, shop, to buy (*e.g. goods*), to own (*possess*), to pay for (*e.g. goods*), to pay (*e.g. in a restaurant*), to sell (*e.g. goods*), ware, wealth
Travel	boat, campfire, east, load (*on a vehicle*), north, oar (*rowing implement*), ski, sleigh, south, step (*single pace*), to drive (*travel by a vehicle*), to row (*propel a boat using oars*), walk (*trip made by walking*), west
Warfare	arrow (*projectile shot from a bow*), bow (*weapon*), enemy (*hostile person*), fight (*physical confrontation*), gun (*firearm*), violence (*use of force*), war, to defend (*e.g. city, state*), to guard (*e.g. a building*), to protect (*a person*), to shoot (*e.g. with a gun*), to win (*achieve victory*)
Writing	book, character (*written symbol, letter*), letter (*written message*), newspaper, stroke (*drawn with a writing implement*), to draw (*e.g. a line*), to paint (*e.g. a picture*), to read (*e.g. a book*), to write (*e.g. a letter*)

## Footnotes

1 http://northeuralex.org.

2 https://zenodo.org/record/1312849#.XFIP-sZCdhE.
